# Visual Impairment among Weaving Communities in Prakasam District in South India

**DOI:** 10.1371/journal.pone.0055924

**Published:** 2013-02-07

**Authors:** Srinivas Marmamula, Saggam Narsaiah, Konegari Shekhar, Rohit C. Khanna

**Affiliations:** 1 Allen Foster Community Eye Health Research Centre, International Centre for Advancement of Rural Eye care, L V Prasad Eye Institute, Hyderabad, India; 2 Bausch & Lomb School of Optometry, L V Prasad Eye Institute, Hyderabad, India; 3 Dana Center for Preventive Ophthalmology, Wilmer Eye Institute, Baltimore, Maryland, United States of America; Zhongshan Ophthalmic Center, China

## Abstract

**Purpose:**

To assess the prevalence and causes of visual impairment in weaving communities in Prakasam district in South India state of Andhra Pradesh.

**Methods:**

Using Rapid Assessment of Visual Impairment (RAVI) methodology, a population based cross-sectional study was conducted. A two-stage sampling strategy was used to select 3000 participants aged ≥40 years. Visual Acuity (VA) was assessed using a tumbling E chart and ocular examinations were performed by trained Para medical ophthalmic personnel. A questionnaire was used to collect personal and demographic information. Blindness and moderate Visual Impairment (VI) was defined as presenting VA <6/60 and <6/18 to 6/60 respectively. VI included blindness and moderate VI.

**Results:**

2848 of 3000 enumerated subjects (94.0%) participated. 39% were in 40–49 years age group and 11.8% were aged ≥70 years, 55% were women and nearly half of them had no formal education. 400 (14%; 95% CI: 12.8–15.3) subjects had VI, including blindness in 131 (4.6%; 95% CI: 3.8–5.4) and moderate VI in 269 (9.4%; 95% CI: 8.3–10.5) individuals. On applying multiple logistic regression, VI was significantly associated with older age and no formal education. Though the odds of having VI were higher in females, it was of borderline statistical significance (p = 0.06). Refractive error was the leading cause of all VI followed by cataract (56%). However, refractive errors were the leading cause of moderate VI (73.2%) and cataract was the leading cause of blindness (62.6%). ‘Cannot afford the cost of services’ was the leading barrier for utilization of eye care services (47%).

**Conclusions:**

There is a significant burden of VI in weaving communities in Andhra Pradesh, India most of which is avoidable. With this information as baseline, services need to be streamlined to address this burden.

## Introduction

Globally, over 285 million people are visually impaired, including 39 million blind and 246 million with low vision. [1] Uncorrected refractive errors and cataract remain the leading causes of visual impairment contributing to over 75% of the total visual impairment. [2]

Studies using rapid assessment methods provide estimates on the burden of visual impairment. These evaluations also provide data for planning eye care services in regions where they are conducted. [3] Rapid assessment survey methods though initially developed for cataract alone, continue to be modified to include assessment of emerging conditions like diabetic retinopathy, refractive errors and presbyopia. [4], [5] They continue to play an important role in blindness prevention programmes.

Cloth weaving is an important industry in Prakasam district of South India state of Andhra Pradesh. [6] Workers use handlooms and the process includes use of different yarns or threads interlaced at right angles to make the cloth. Typically the entire family is involved and the older individuals mostly perform skill-based activities. The weaving of cloth demands good vision for distance and near as process involves spinning yarn or threads and use of fine tools for patterns on the cloth. Visual impairment limits worker productivity and may affect the economic status of the family. We report on the prevalence and causes of visual impairment and blindness using a low-cost rapid assessment methodology called ‘Rapid Assessment of Visual Impairment (RAVI)’ among those aged 40 years and older in weaving communities in Prakasam district. [4]

## Methodology

### Ethics Statement

The study protocol was reviewed and approved by Institutional Review board (IRB) (Scientific and Ethics committee) of Hyderabad Eye Research Foundation, L V Prasad Eye Institute, Hyderabad, India in June 2010. The study was conducted in accordance with the tenets of the Declaration of Helsinki.

Permission was obtained from head of the each village before starting the data collection. At household level, the study procedures were explained to each individual and Oral consent was obtained in presence of other family members and another individual who does not belong to same family, usually a neighbor. Each individual was free to decide on participation in the study.

IRB gave the approval for oral consent for two reasons; a) The study procedures were non invasive and did not involve any physical contact with the subjects or administration of any medication. The procedures are a part of regular eye screening protocols used at primary level in India and b) The literacy levels in the geographical location where the study was conducted was not very high and general apprehension in the community towards providing thumb impression on the consent form, which in turn may lead to poor response rate. The provision of verbal consent was documented by taking photographs to illustrate the process in selected villages and by random visits to the villages by the Principal investigator for cross verification with the participants as a part of the quality control measures.

### Study area

The population of the Prakasam district was estimated at 3.0 million in 2001, with an annual growth rate of 1.08%. [7] This district is divided into 56 mandals (sub districts or administrative divisions). The study was conducted in four administrative divisions predominantly inhabited by weaving communities. The total population of this region was 335,509 as per 2001 census.

### Sample size

Sample size was determined using a prevalence estimate of 5% blindness in those 40 years of age and older. Allowing for a 95% confidence interval, a precision of 20%, design effect of 1.5 for a predetermined cluster size of 50 subjects and 10% non-response rate, the sample size required was 3000 (60 clusters). As per Andhra Pradesh Eye Disease Study, the prevalence of blindness among those aged 40 years and older was 8.4%. Considering about 3% decrease in prevalence of blindness since the time of APEDS due to improved service delivery, a prevalence estimate of 5% was used for sample size calculation. [8]

Villages predominantly inhabited by weaving communities were identified and listed. Larger villages were divided into smaller clusters in such a way that the population in each cluster was similar in size. From this list, 60 clusters were randomly selected and from each cluster, attempts were made to enumerate and examine 50 individuals aged 40 years and older. Once a cluster is identified, individual households are selected using Expanded Programme on Immunization (EPI) random walk method. [9] Repeated attempts were made to examine the subjects who were not available at the time of the first visit. Subjects who were not available after two attempts and those who refused to participate were not substituted.

### Study teams

Three field teams were involved in the data collection. Each team consisted of an experienced vision technician (with at least 3 years of experience in primary eye care) along with one community eye health worker. Vision Technicians are the personnel trained for one year to provide primary eye care including visual acuity assessment, screening for potentially blinding eye conditions, refraction and dispensing of spectacles in rural areas. The teams underwent two days of training in the study procedures. The training covered all aspects related to selection of clusters, enumeration methods, clinical examination, coding and data entry, and maintenance of daily records. Inter observer reliability assessment was done with a senior optometrist (gold standard) for the distance and near vision assessments. A kappa value of 0.7 was considered as adequate.

### Data collection process

The subjects were visited in their homes by the survey team. Detailed examination protocol is described elsewhere. [4] In brief, unaided (and aided) visual acuity (VA) in each eye was measured using a Snellen chart with tumbling “E” optotypes at a distance of 6 meters. VA was assessed in shade during the daytime on sunny days. Due precautions were taken to avoid reflections and glare on the VA chart. Subjects with VA less than 6/18 in either eye were re-assessed using a multiple pinhole occluder. Near vision was assessed binocularly using the N notation chart at a fixed distance of 40 cm for each individual. Both unaided and aided near vision were assessed if the subject reported using spectacles. Near vision was re-assessed among subjects who had near vision <N8 by using near addition lenses appropriate for that age. Torchlight examination was performed to assess the anterior segment of the eye. Lens status was assessed by using a torchlight and distant direct ophthalmoscopy in a shaded environment without pupillary dilatation.

Demographic information including education level, occupation, current and previous use of spectacles was collected through a brief personal interview. A question on barriers to uptake of eye care services was asked of all subjects with visual impairment. The examiner had a list of possible responses. If the response/s reported by the subject was on the list, then it was marked. In cases where one or more barriers reported were not in the list, they were fully specified under ‘others’. The subject was also asked to specify the single most important barrier, and this was used in the analysis. All subjects with visual impairment or those who needed eye care services were provided with a referral letter to visit the nearest eye care facility for management.

### Study definitions

Indian definitions for blindness and moderate visual impairment were used to facilitate comparison with previous studies. [10], [11] Any visual Impairment (VI) was defined as presenting VA <6/18 in the better eye. Moderate visual impairment (MVI) and blindness were defined as presenting VA <6/18 to 6/60 and <6/60 in the better eye, respectively. The Indian definition of blindness includes the category of Severe VI and blindness as defined by the WHO. Refractive Error was defined as presenting VA <6/18, but improving to 6/18 or better with pinhole. Cataract was defined as opacity of the crystalline lens in the pupillary area as seen with a torchlight through an undilated pupil and resulting in presenting VA <6/18 and not improving with pinhole. Posterior segment disease was considered as present if there was no improvement in VA on using a pinhole, and no obvious media opacity on torch light examination.

In cases where there was more than one cause for VI, the one which was more easily treatable or correctable to achieve a VA ≥6/18 was considered as the primary cause of VI. For example, if a patient had an operable cataract and refractive error, the cause was marked as refractive errors as it is easier to correct it compared to the surgical intervention for cataract. This is the convention used in population based surveys as per recommendation of World Health Organization. [12]

### Data management

Data analysis was conducted using SPSS 16.0 (SPSS Inc., Chicago, IL). Point prevalence estimates and 95% confidence intervals (CI) were calculated. Statistical significance was assessed by chi-square test for categorical variables and t test for continuous variables. Multiple logistic regression analysis was used to assess the strength of association between VI and other personal risk factors (age, gender and level of education). Odds ratio with 95% CI is reported. P value of <0.05 was considered as statistically significant.

## Results

**Figure 1:**
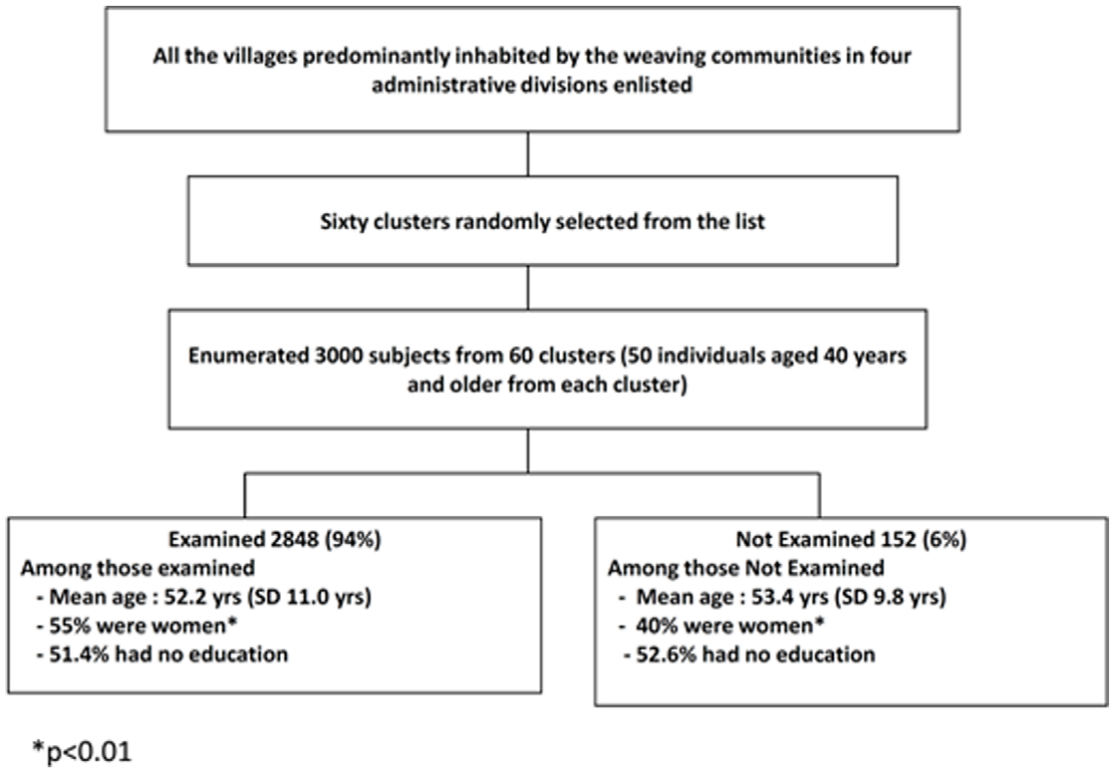
Flow chart showing the sampling process and baseline characteristics of the sample.

2848 of 3000 enumerated subjects (94.0%) participated in the study. Among 152 individuals who were not examined, 91 (60%) were male and 108 (71%) were less than 60 years of age Mean age and level of education of those examined and not examined were statistically similar. More women are likely to be available for examination compared to men (chi squared test; p<0.01). The sampling procedure and demographic characteristics are shown in Figure 1 (Figure 1). Among those examined, 39% were in 40–49 years age group and 11.8% were aged 70 years and older, 55% were women and nearly half of them had no formal education (Table 1). Over 95% of those currently working were involved in weaving or weaving related activities.

**Table 1:** Participants Characteristics and categories of Visual Impairment.

	All participants (n = 2848)	No Visual Impairment (n = 2448)	Moderate Visual Impairment * (n = 269)	Blindness# (n = 131)	Any Visual Impairment ## (n = 400)	Adjusted Odds ratio (95% CI)
**Age Group (yrs)**						
40–49	1101	1065 (96.7)	28 (2.5)	8 (0.7)	36 (3.3)	1.0
50–59	800	718 (89.8)	61 (7.6)	21 (2.6)	82 (10.3)	3.5 (2.3–5.2)
60–69	612	471 (77.0)	89 (14.5)	52 (8.5)	141 (23.0)	8.7 (5.9–12.7)
70 and above	335	194 (57.9)	91 (27.2)	50 (14.9)	141 (42.1)	22.4 (15.0–33.5)
**Gender**						
Male	1283	1127 (87.8)	105 (8.2)	51 (4.9)	156 (13.1)	1.0
Female	1565	1321 (84.4)	164 (10.5)	80 (5.1)	244 (15.6)	1.3 (1.0–1.7)
**Education Level**						
No formal education	1465	1202 (82.0)	174 (11.9)	89 (6.1)	263 (18.0)	1.0
Any education	1383	1246 (90.1)	95 (6.9)	42 (3.0)	137 (9.9)	1.7 (1.3–2.2)

* *Moderate Visual Impairment is defined as presenting visual acuity worse than 6/18 to 6/60 in the better eye.*

# *Blindness is defined as presenting visual acuity worse than 6/60 in the better eye.*

## *Any visual impairment includes both the blindness and moderate visual impairment; Visual impairment categorized as yes or no.*

In total, 400 (14%; 95% CI: 12.8–15.3) subjects had VI, including blindness in 131 (4.6%; 95% CI: 3.8–5.4) and moderate VI in 269 (9.4%; 95% CI: 8.3–10.5) individuals. On applying multiple logistic regression, VI was significantly associated with older age and no formal education. Though the odds of having VI were higher in females, it was of borderline statistical significance (p = 0.06). Refractive error was the leading cause of all VI followed by cataract (56%). However, refractive errors were the leading cause of moderate VI (73.2%) and cataract was the leading cause of blindness (62.6%) (Table 2). Based on the WHO definition (presenting VA <3/60 in the better eye), the prevalence of blindness was 2.9% (95% CI: 2.3–3.5).

**Table 2:** Causes of Visual Impairment (n = 400).

	Moderate Visual Impairment * n (%)	Blindness n (%)#	All Visual Impairment n (%)##
Refractive error	197 (73.2)	27 (20.6)	224 (56.0)
Cataract	50 (18.6)	82 (62.6)	132 (33.0)
Uncorrected Aphakia	12 (4.5)	8 (6.1)	20 (5.0)
Cataract surgical complications	3 (1.1)	6 (4.6)	9 (2.3)
Posterior segment disease	6 (2.2)	5 (3.8)	11 (2.8)
Others	1 (0.4)	3 (2.3)	4 (1.0)
	269 (100.0)	131 (100.0)	(100.0)

* *MVI is defined as presenting visual acuity worse than 6/18 to 6/60 in the better eye.*

# *Blindness is defined as presenting visual acuity worse than 6/60 in the better eye.*

## *Any visual impairment includes both the blindness and moderate visual impairment; p<0.05.*

The leading barrier for utilization of eye care was economic concerns among those with moderate VI and blindness, and was reported by 47% of both groups. Other stated primary barriers to obtaining eye care was lack of ‘Felt need’ (28.3% and 33.6%), personal reasons (17.8% and 12.2%) and service related barriers (4.1% and 7.6%) among those with moderate VI and blindness, respectively. (Table 3)

**Table 3:** Barriers for uptake of services among visually impaired (n = 400).

Main Barrier	Moderate Visual Impairment (<6/18–6/60) n (%)	Blindness (<6/60) n (%)	All Visual Impairment (<6/18) n (%)
Cannot afford cost of services (E)	126 (46.8)	61 (46.6)	187 (46.8)
Aware of the problem, need not felt (FN)	43 (16.0)	18 (13.7)	61 (15.3)
Old age need not felt (FN)	33 (12.3)	24 (18.3)	57 (14.3)
One eye vision adequate (FN)	8 (3.0)	2 (1.5)	10 (2.5)
No time, other commitments (P)	17 (6.3)	3 (2.3)	20 (5.0)
Other health problems (P)	11 (4.1)	5 (3.8)	16 (4.0)
No escort (P)	9 (3.3)	5 (3.8)	14 (3.5)
Fear of losing sight(P)	4 (1.5)	3 (2.3)	7 (1.8)
Unaware of the problem (P)	7 (2.6)	0 (0.0)	7 (1.8)
Waiting for cataract to mature (S)	9 (3.3)	8 (6.1)	17 (4.3)
Services very far (S)	2 (0.7)	2 (1.5)	4 (1.0)
	**269 (100.0)**	**131 (100.0)**	**400 (100.0)**

*E = Economic reasons; FN = Barriers related to felt need; P = Personal reasons; S = Service related barriers.*

## Discussion

The current study documented a prevalence of blindness and moderate VI of 4.6% and 9.4% respectively in persons aged 40 year and older living in a weaving community in rural India. Those over the age of 50 years had a slightly lower prevalence of blindness and moderate VI to what was reported in a recent Rapid Assessment of Avoidable Blindness (RAAB) national survey in India. [11] Possible explanations include greater availability of services in this area and differences in methodology and other reasons related to health seeking behavior and visual needs

The prevalence was also substantially lower than that was found in a previous study using the same study protocol in fishing communities from the same district. In that study the prevalence of blindness and moderate VI were 7.1% and 22.8%, respectively. [4] This difference can partly be explained by the geographical distribution of the populations studied and availability of services in their regions. The fishing communities reside along the coast in small settlements whereas the weaving communities studied were located closer to the urban locations where the eye care services can be accessed. It is also possible that the socio economic reasons and the visual demands could be contributing factors for this difference.

Similar to other studies done in India, Sri Lanka, Pakistan, Nepal and Bangladesh, cataract and uncorrected refractive errors were the leading causes of VI, though there are differences in the age groups studied and the study methods used. [11], [13]–[18] Reports from India on association between VI and female gender have been inconsistent. [4], [8], [11], [19] Though we found a positive association between the gender and VI, it was of borderline statistical significance.

The lack of affordability continues to remain as the leading barrier preventing the uptake of eye care services. This was similar to the findings reported over a decade ago. [20] Barriers related to lack of felt need and personal reasons were also reported from Sri Lanka and Nepal. [14], [21] In Sri Lanka, was lack of desire to improve vision, fear of surgery and lack of awareness were the barriers whereas in Nepal, affordability, lack of awareness about the treatment and non availability of escort were the important barriers. [14], [21]

Provision of service at ‘no cost’ can help address this issue related to affordability to some extent. The National Programme for Control of Blindness provides subsidy for free cataract surgeries done by non government institutions in India. But the ‘indirect costs’ of procuring services remains an issue to be addressed. Barriers related to ‘felt need’ and personal reasons are more difficult to address than economic barriers. Health education campaigns highlighting the advances in cataract surgical techniques, quick healing time without significant restriction on activities of daily leaving, faster visual recovery can help a significant proportion of people to undergo cataract surgery. Comprehensive eye care services should not only include affordable and accessible care but it also need to effectively address the above mentioned barriers through eye health education in the communities.

This is the first study to report on the burden of VI among cloth weaving communities in Andhra Pradesh, India. Although the current study was conducted in Prakasam District, the results may be extrapolated to the rest of weaving communities in the entire state as living conditions and the geographical distribution have little variations. This community has been facing difficult times since few years leading to suicides as a result of debt burden. [6] Several weavers have changed their profession but significant proportion of people still in this profession as this is the only work they know from generations. [6] This study is designed to understand the burden of visual impairment in this community to formulate the provision of eye care services in this region.

Over 90% of the sampled population participated indicating that the results are likely representative of the larger weaving population. It is possible that we overestimated cataract as a cause of vision loss since we did not perform dilated eye examination. Other limitation is that we used torchlight to assess the anterior segment of the eye.

With the information on the burden of VI now available, it is now the time to plan effective strategies to address this burden by providing good quality eye care services coupled with comprehensive health education programmes addressing the issues related to uptake of services.
